# Complexity-Entropy Characterization of Storage Dynamics in Semiarid Reservoirs: Linking Ordinal Patterns with Elevation-Storage Curve Shifts in Paraíba, Brazil

**DOI:** 10.3390/e28070779

**Published:** 2026-07-08

**Authors:** Ana Kerma Araujo Gomes de Sousa, Laércio Leal dos Santos, Fernando Henrique Antunes de Araujo

**Affiliations:** 1 Department of Civil Engineering, Mathematics and Statistics, Federal Institute of Education, Science and Technology of Paraíba (IFPB), Patos 58700-030, Brazil; ana.kerma@academico.ifpb.edu.br; 2 Department of Sanitary and Environmental Engineering, State University of Paraíba (UEPB), Campina Grande 58429-500, Brazil; laercioleal@servidor.uepb.edu.br

**Keywords:** permutation entropy, statistical complexity, complexity-entropy causality plane, reservoir storage, hydrological time series, semiarid reservoirs, elevation-storage curves, monitoring

## Abstract

Hydrological reservoirs are complex systems in which storage variations integrate climate forcing, catchment response, releases, withdrawals, evaporation, and monitoring procedures. This study presents an information-theoretic characterization of storage dynamics in five semiarid reservoirs in Paraíba, Brazil. The main analytical layer is the complexity–entropy causality plane (CECP), computed from daily storage increments by permutation entropy and Martín-Plastino-Rosso statistical complexity. CECP was estimated for fixed periods and for sliding windows of 120 observations, with sensitivity tests for embedding dimension, delay, and window length. The workflow also benchmarks CECP distance against conventional descriptors, quantifies ties and zero increments, and tests window overlap. As physical context, monotonic elevation-storage curves were reconstructed for 2009–2014, 2015–2019, and 2020–2026, and storage differences at equivalent water levels were quantified by bootstrap confidence intervals. The reservoirs occupied a high-entropy, low-to-moderate-complexity region of the CECP, but their distances from the maximum-entropy/minimum-complexity vertex differed across reservoirs and periods. Sliding windows revealed temporal mobility that was hidden by fixed-period summaries, especially in Engenheiro Arcoverde, Jatobá I, and Mãe d’Água. Rankings remained strongly concordant when window overlap decreased from 94.2% to 0% (Spearman ρ=0.943), although absolute coordinates were sensitive to the treatment of reported plateaus. Elevation-storage shifts provided an independent structural context: negative shifts were compatible with possible useful-capacity reduction, although not uniquely attributable to sedimentation. The results show that CECP descriptors can reveal ordinal organization and regime mobility in reservoir storage increments, while V(H) curves supply the physically interpretable storage-capacity context. The combined evidence prioritizes Engenheiro Arcoverde and Mãe d’Água for bathymetric, curve history, and operational verification. The proposed workflow is therefore an exploratory information-theoretic screening tool for data-limited reservoir monitoring, not a substitute for bathymetric validation.

## 1. Introduction

Reservoir storage dynamics are produced by the interaction of hydrological forcing, catchment response, evaporation, releases, withdrawals, and monitoring practice. In semiarid regions, these processes are amplified by strong rainfall intermittency, high evaporative demand, drought recurrence, and operational pressure on limited surface-water reserves [1,2,3]. For this reason, reservoir time series are not merely engineering records. They are empirical signatures of a complex water system whose temporal organization can change across climatic and operational regimes.

Changes in reservoir capacity are commonly evaluated through repeated bathymetric or hydrographic surveys, sediment sampling, sediment-yield and transport models, empirical trap-efficiency relations, or remote-sensing reconstructions of surface area and water level [4,5,6,7,8,9,10,11]. Bathymetry provides the most direct evidence of geometric change, but repeated surveys can be expensive and infrequent. Recent reservoir-sedimentation studies reinforce the engineering relevance of this problem by documenting capacity loss and trapping-efficiency changes with bathymetric, GIS, and field-based approaches [6,7]. Remote sensing and simplified empirical methods improve spatial and temporal coverage, yet their accuracy depends on image availability, shoreline detection, water-level information, and local calibration. Operational elevation and storage records are more widely available, but they are indirect because official curve revisions, datum changes, sensor recalibration, interpolation procedures, and reporting practices can also alter the empirical elevation-storage relationship.

### 1.1. Related Work and Positioning

Information-theoretic methods are well suited to this type of problem because they characterize organization, uncertainty, and temporal order without requiring a complete mechanistic model. Entropy-based approaches have a long history in hydrology and water engineering. Previous work in Entropy has discussed the relationship between thermodynamic and probabilistic views of hydrological inference, entropy and copula concepts for hydrologic dependence modeling, monitoring-network design using ensemble entropy, entropy-based approaches to discharge estimation, soil-moisture modeling, and information-theoretic analyses of river-flow regimes [12,13,14,15,16,17]. Recent stochastic and information-oriented hydrological studies further show how probabilistic prediction, streamflow uncertainty, and ordinal information quantifiers can support interpretation of nonstationary water-system dynamics [18,19,20,21]. These studies show that entropy methods can provide useful descriptions of hydrological variability, uncertainty, and scale-dependent organization.

The complexity–entropy causality plane (CECP) is a particularly useful representation for empirical time series. It combines normalized permutation entropy, introduced by Bandt and Pompe [22], with statistical complexity based on the Martín-Plastino-Rosso framework [23]. Permutation entropy maps a time series into ordinal patterns, while statistical complexity measures the coexistence of entropy and structured departure from equiprobability. The theoretical and practical literature has established the CECP representation and documented choices involving parameterization, ties, and amplitude sensitivity [24,25,26,27]. Applications have subsequently distinguished stochastic, chaotic, and empirical regimes in several complex systems [28,29,30], including recent hydrological applications [20,21].

The practical use of permutation quantifiers nevertheless requires explicit choices. Embedding dimension and delay determine the ordinal alphabet and temporal scale; short samples can undersample the D! possible patterns; overlapping windows generate dependent estimates; and ties caused by finite measurement or reporting precision require a stated convention [25,26,27]. These issues are especially relevant for operational reservoir series, in which unchanged reported storage can represent genuine short-term stability, finite precision, delayed reporting, or a combination of these factors. Parameter and reporting sensitivities must therefore accompany physical interpretation.

The present study applies the CECP to daily storage increments in semiarid reservoirs. The central information-theoretic question is whether reservoirs that are observed under the same regional climate occupy similar or different entropy-complexity domains, and whether their ordinal organization changes through time. This question is deliberately different from direct sedimentation estimation. Storage increments integrate inflows, outflows, rainfall over the water surface, evaporation, withdrawals, and measurement/reporting effects. CECP descriptors can therefore characterize the dynamics of the monitored storage response, but they cannot identify a single physical cause for those dynamics.

To provide a physically interpretable storage-capacity context, we also reconstruct monotonic elevation-storage curves, V(H), for fixed periods. These curves relate water level to stored volume. If a more recent curve stores less water at the same water level over a common support, the shift is compatible with useful-capacity reduction and may justify field verification. Similar storage-capacity questions have been addressed through semiarid-reservoir studies, bathymetric surveys, and hydrographic or remote-sensing approaches [4,5,6,7,8,9,10,11]. However, this structural evidence is indirect and non-unique: official curve revisions, datum changes, sensor recalibration, monitoring practice, and database updates may produce similar shifts.

Conventional time series descriptors provide a transparent baseline. The mean and standard deviation characterize location and amplitude, median-based measures provide robust summaries of magnitude and dispersion, skewness describes asymmetry, lag-1 autocorrelation measures linear persistence, and Spearman correlation provides a rank-based comparison [31,32,33]. Each statistic targets a specific property. In contrast, the Bandt-Pompe distribution aggregates the relative frequencies of multistep temporal orderings. A rigorous application must therefore test whether CECP coordinates add information beyond those simpler summaries rather than merely assuming that they do.

The methodological gap addressed here is consequently narrower than direct capacity estimation. Data-limited agencies need a transparent screening procedure that indicates where dynamical behavior, reported storage geometry, or both have changed enough to justify closer investigation. Such a procedure must show what information CECP contributes beyond simpler statistics, quantify its sensitivity to analytical choices, and convert the joint evidence into follow-up actions without overstating physical attribution.

### 1.2. Objective and Contributions

The objective of this article is therefore to develop an Entropy-oriented workflow for reservoir monitoring that combines: (i) conventional descriptors and CECP quantifiers of storage increment dynamics; (ii) sliding-window CECP trajectories that reveal temporal mobility; (iii) elevation-storage curve shifts as physical context for possible capacity change; and (iv) a sample-relative decision matrix for prioritizing bathymetric, operational, curve history, or routine monitoring actions. We hypothesize that CECP quantifiers contain ordinal information not captured by variance or linear autocorrelation, while V(H) shifts provide a distinct structural context. The contribution is not a validated siltation diagnostic. It is an exploratory information-theoretic framework for ranking and interpreting reservoir storage dynamics in data-limited systems. External bathymetric validation is unavailable; accordingly, the study evaluates internal robustness and operational interpretability rather than diagnostic accuracy for sedimentation.

## 2. Study Area and Data

### 2.1. Semiarid and Spatial Context

The study area is located in Paraíba, northeastern Brazil. Four selected reservoirs are situated in the Sertão domain and one in the Cariri transition. These environments are characterized by rainfall concentrated in a short wet season, high interannual variability, recurrent drought, and dry-season evaporative losses [1,2,3]. Most studied reservoirs belong to the Piancó-Piranhas-Açu system; Sumé belongs to the Upper Paraíba system. Their contrasting capacities and storage ranges provide a useful, although geographically restricted, test set.

**Figure 1:**
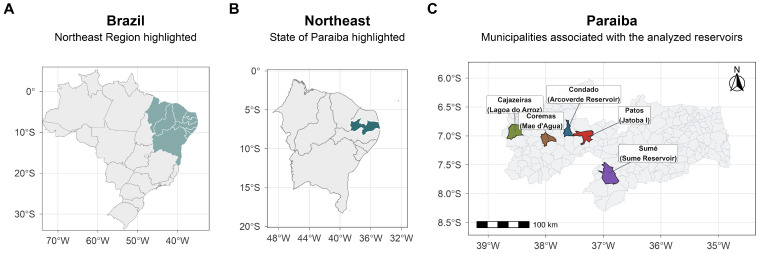
Study area and spatial context of the selected reservoirs. (**A**) Location of Brazil in South America. (**B**) Location of the Northeast region within Brazil. (**C**) Location of Paraíba within the Northeast region, and municipalities associated with the selected reservoirs in Paraíba, highlighting Condado, Patos, Cajazeiras, Coremas, and Sumé.

The nested geographic context of the study area, from South America to the municipalities associated with the selected reservoirs, is shown in Figure 1.

**Figure 2:**
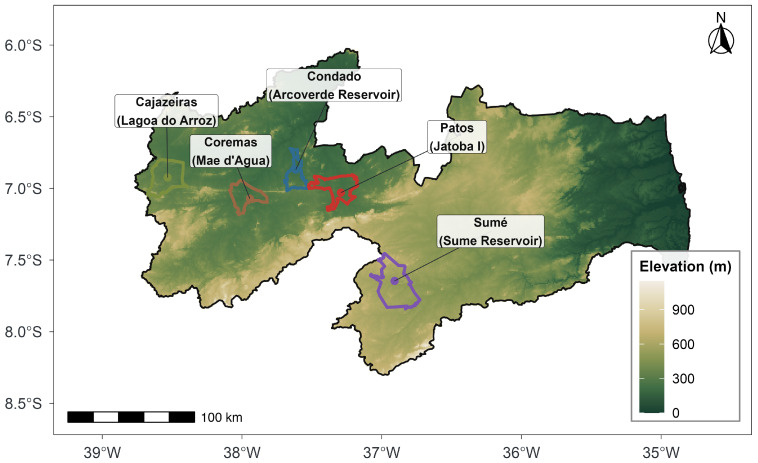
Hypsometric map of Paraíba and municipalities associated with the selected reservoirs.

The elevation setting and the location of the selected municipalities within Paraíba are shown in Figure 2.

Table 1 presents reservoir location, nominal capacity, monitoring coverage, and observed elevation-storage domains. These quantities describe the empirical support of the analysis; they are not bathymetric measurements.

### 2.2. Data Source, Availability, and Interpretation Boundary

The operational time series of date, water level H, and stored volume V were obtained from the reservoir monitoring system of the Executive Water Management Agency of the State of Paraíba (AESA), accessed in 2026 [34]. A record entered the paired H-V analysis only when date, positive water level, and positive stored volume were simultaneously available. No observation with a missing member of the pair was imputed. The CECP series was computed from chronologically ordered valid storage records and normalized by the elapsed number of days.

The AESA file does not contain reservoir-specific rainfall, inflow, evaporation, releases, withdrawals, land use, sediment concentration, repeated bathymetry, or a documented history of official V(H) revisions. Table 2 states how the main physical controls enter the present analysis. The absence of these variables prevents causal decomposition and external validation. It does not prevent descriptive analysis of the reported storage response, but it limits the admissible inference.

### 2.3. Reservoir Selection and Period Definition

The source database contained ten reservoirs. To ensure a homogeneous application, reservoirs were retained only when all three fixed periods contained at least 120 valid paired observations and all three pairwise period comparisons had a nonempty common water-level interval. Five reservoirs satisfied these criteria. The fixed periods were 2009–2014 (P1), 2015–2019 (P2), and 2020–2026 (P3). They provide comparable analytical blocks rather than causal climatic phases. The complete record was analyzed separately for an overall CECP position.

The full selection audit is reported in Appendix A, Table A1. This audit avoids selecting reservoirs on the basis of the resulting CECP coordinates or V(H) differences.

## 3. Methods

### 3.1. Analytical Workflow

**Figure 3:**
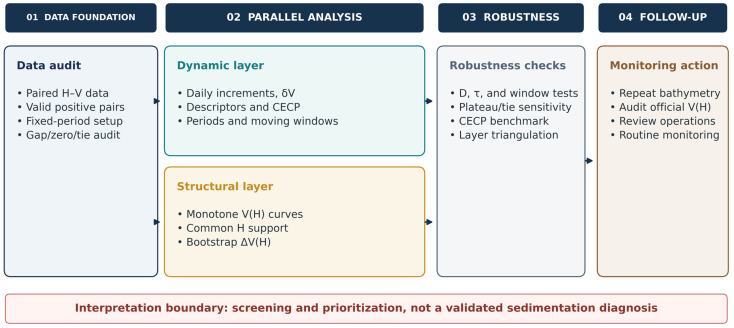
Methodological workflow. CECP and V(H) evidence support screening and prioritization, not a validated diagnosis of sedimentation.

Figure 3 summarizes the sequence from data audit to follow-up recommendation. The dynamic and structural layers are calculated independently and joined only during interpretation. Robustness checks are applied before a reservoir is assigned to a sample-relative follow-up category.

### 3.2. Storage Increments and Conventional Descriptors

For two consecutive valid reports, the storage change was

(1) $$\Delta V_{t}=V_{t}-V_{t-1},$$

and the daily normalized increment was

(2) $$\delta V_{t}=\frac{\Delta V_{t}}{\Delta d_{t}},$$

where $\Delta d_{t}$ is the elapsed number of days. This normalization prevents a multi-day reporting interval from being interpreted as a one-day change. No interpolation was used to manufacture daily observations.

The reported increment can be represented schematically as

(3) $$\delta V_{t}\approx Q_{in,t}-Q_{out,t}+P_{t}A_{t}-E_{t}A_{t}-W_{t}+\eta_{t},$$

where $Q_{in,t}$ and $Q_{out,t}$ are inflow and controlled outflow, $P_{t}A_{t}$ and $E_{t}A_{t}$ are direct rainfall and evaporation over the water surface, $W_{t}$ denotes withdrawals, and $\eta_{t}$ aggregates measurement, rounding, and reporting effects. Because the right-hand components are unavailable separately, CECP is interpreted as a descriptor of their integrated reported response.

Let $x_{i}=\delta V_{i}$, i=1,…,n, and let $\tilde{x}$ denote the sample median. The location and scale descriptors were calculated as

(4) $$\bar{x}=\frac{1}{n}\sum_{i=1}^{n}x_{i},\;\;s_{x}=\sqrt{\frac{1}{n-1}\sum_{i=1}^{n}(x_{i}-\bar{x}{)}^{2}},\;\;M_{\left|x\right|}=median(|x_{i}|),\;\;{MAD}_{x}=median(|x_{i}-\tilde{x}|),$$

where $M_{\left|x\right|}$ measures the typical absolute daily change and ${MAD}_{x}$ is a robust measure of dispersion [31]. Sample skewness and lag-1 autocorrelation were

(5) $$g_{1}=\frac{n}{\left(n-1)(n-2\right)}\sum_{i=1}^{n}{\left(\frac{x_{i}-\bar{x}}{s_{x}}\right)}^{3},\;\;r_{1}=\frac{\sum_{i=2}^{n}(x_{i}-\bar{x})(x_{i-1}-\bar{x})}{\sum_{i=1}^{n}(x_{i}-\bar{x}{)}^{2}},$$

with the finite-sample skewness correction following Joanes and Gill [33]. Reporting plateaus and directional balance were summarized by

(6) $$f_{0}=\frac{1}{n}\sum_{i=1}^{n}I(|x_{i}|\leq \varepsilon ),\;\;f_{+}=\frac{1}{n}\sum_{i=1}^{n}I(x_{i}>\varepsilon ),$$

where ε is the numerical zero tolerance. We also recorded the longest consecutive run satisfying $|x_{i}|\leq \varepsilon$. Spearman correlations,

(7) $$\rho_{s}=Corr\{R(E),R(Z)\},$$

were calculated between CECP distance E and each conventional descriptor Z, using average ranks for ties [32]. This rank-based redundancy benchmark asks whether E behaves as a monotonic surrogate for an individual statistic. A weak correlation does not establish superior predictive performance; it only indicates that the two summaries are not monotonically interchangeable in this sample.

### 3.3. Permutation Entropy and Statistical Complexity

For a series $X=\{x_{t}\}$, the embedding vector was

(8) $$Y_{s}=[x_{s},x_{s+\tau },x_{s+2\tau },\ldots ,x_{s+(D-1)\tau }],$$

where D is the embedding dimension and τ is the delay. Each vector was mapped to the permutation π that orders its components. The relative frequencies of the D! ordinal patterns define $P=\{p_{i}\}$. Normalized permutation entropy was calculated as [22]

(9) $$H[P]=-\frac{\sum_{i=1}^{D!}p_{i}lnp_{i}}{ln(D!)},$$

with 0≤H≤1. Statistical complexity was [23]

(10) $$C[P]=Q_{J}[P,P_{e}]H[P],$$

where $P_{e}$ is the uniform distribution and $Q_{J}$ is the normalized Jensen-Shannon disequilibrium based on

(11) $$J[P,P_{e}]=S\left[\frac{P+P_{e}}{2}\right]-\frac{S[P]}{2}-\frac{S[P_{e}]}{2}.$$

The CECP retains the pair (H,C) as the primary representation. For temporal summaries, we additionally used

(12) $$E=\sqrt{(1-H{)}^{2}+C^{2}},$$

the Euclidean distance to the maximum-entropy, minimum-complexity vertex (1,0). The index is justified as a compact radial coordinate for tracking movement relative to that reference corner. It combines both CECP axes and supports ranking or sliding-window visualization. However, it is not unique: different (H,C) pairs can yield the same E, and equal weighting of the two normalized axes is a descriptive choice. Consequently, E is always reported with H and C for fixed periods and is never interpreted as hydrological efficiency, management quality, or sedimentation intensity.

### 3.4. Embedding Dimension, Sample Size, and Ties

The primary configuration was D=4 and τ=1, giving 24 ordinal patterns. The minimum series length followed the conservative rule [35]

(13) $$N_{min}=5D!,$$

which gives 120 observations for D=4. The D=5 sensitivity requires at least 600 observations and was therefore exploratory where this condition was satisfied. The alternative τ=2 tested a longer ordinal delay.

Equal values were handled by the deterministic convention of the statcomp ordinal-pattern implementation used for all primary calculations. This choice preserves reported plateaus as part of the monitored signal. If M=N−(D−1)τ is the number of embedding vectors and $u(Y_{s})$ is the number of distinct values in vector $Y_{s}$, the tied-vector fraction was

(14) $$f_{tie}=\frac{1}{M}\sum_{s=1}^{M}I\{u(Y_{s})<D\}.$$

To expose the consequences of ties, two complementary checks were applied. First, exact zero increments were excluded to form the event-only series $X_{nz}=\{x_{i}:|x_{i}|>\varepsilon \}$. Second, infinitesimal random jitter was applied 200 times to each fixed-period series:

(15) $$x_{i}^{\left(b\right)}=x_{i}+u_{i}^{\left(b\right)},\;\;u_{i}^{\left(b\right)}∼U(-a,a),\;\;b=1,\ldots ,200,$$

where a was above the implementation tolerance but below the reporting resolution. The jitter experiment is an intentionally extreme perturbation because it converts plateaus into random orderings; it defines a sensitivity boundary rather than a physically preferred correction. Absolute CECP coordinates and rankings that depend strongly on this perturbation are interpreted cautiously [25,26,27].

### 3.5. Fixed-Period and Sliding-Window CECP

CECP quantifiers were first estimated for the complete record and each fixed period. Temporal mobility was then evaluated using windows of W=120 observations advanced by s=7 observations. Windows crossing period boundaries were excluded. The percentage overlap between consecutive windows was

(16) $$O(W,s)=100max\left(0,\frac{W-s}{W}\right).$$

Thus, the primary setting produces 94.2% overlap. Adjacent estimates are strongly dependent and are not treated as independent inferential replicates. Their means and standard deviations are descriptive summaries of a smooth trajectory.

Window lengths of 180 and 240 observations tested the effect of increased smoothing. Steps of 30, 60, and 120 observations tested 75%, 50%, and 0% overlap while keeping the 120-observation window fixed. Stability was assessed using Spearman rank correlation across the 15 reservoir-period cases.

### 3.6. Elevation-Storage Curves

For reservoir r and period p, valid observations were denoted by $\left\{(H_{i},V_{i})\right\}$. Water levels were rounded to 0.1 m classes $h_{j}$, and the conditional median storage was

(17) $$\tilde{V}(h_{j})=median\{V_{i}:H_{i}\in h_{j}\}.$$

The median reduces the influence of local scatter. Isotonic regression [36] imposes the physical nondecreasing condition

(18) $$V_{r}^{\left(p\right)}(H_{j+1})\geq V_{r}^{\left(p\right)}(H_{j}),\;\;H_{j+1}>H_{j}.$$

For periods a and b, comparisons were restricted to the common elevation interval $I_{ab}$. At each common grid elevation,

(19) $$\Delta V(H_{k})=V_{b}(H_{k})-V_{a}(H_{k}),$$

and the average shift was

(20) $$\bar{\Delta V}=\frac{1}{m}\sum_{k=1}^{m}\Delta V(H_{k}).$$

Nonparametric bootstrap resampling of the grid-level differences produced 95% confidence intervals and two-sided bootstrap probabilities [37]. The geometric motivation is

(21) $$V(H)=\int_{0}^{H}A(z)\;dz,$$

where A(z) is the inundated area at elevation z. A negative ΔV(H) is compatible with a reduction of the effective storage domain below H. It is not unique evidence of sediment accumulation because monitoring or official curve changes can produce a similar operational signature.

### 3.7. Robustness and Validation Strategy

Validation was separated into three levels. Internal computational robustness included parameter, window-length, overlap, zero-increment, and tie sensitivities. Statistical uncertainty of V(H) shifts was evaluated by bootstrap. Convergent interpretation compared CECP behavior with conventional descriptors and with the independently reconstructed structural layer. None of these replaces external physical validation.

Repeated bathymetry, sediment surveys, documented rating-curve histories, rainfall, inflow, evaporation, and release data were unavailable. Therefore, sensitivity, specificity, predictive accuracy, and causal attribution for sedimentation cannot be estimated. The word “validation” in this study refers only to internal robustness checks unless explicitly qualified as external validation.

### 3.8. Operational Triangulation

The screening matrix uses continuous evidence before assigning a qualitative action. For reservoir r, structural magnitude, dynamical range, and recent mobility were defined as

(22) $$S_{r}=100\frac{\left|{\bar{\Delta V}}_{r,long}\right|}{K_{r}},\;\;R_{r}=\underset{p}{max}(E_{r,p})-\underset{p}{min}(E_{r,p}),\;\;M_{r}=SD\{E_{r,w}:w\in P3\},$$

where $K_{r}$ is nominal capacity, p indexes the three fixed periods, and w indexes sliding windows in the recent period. “Higher” and “lower” flags were assigned by comparing $S_{r}$ and the joint dynamical evidence $\left(R_{r},M_{r}\right)$ with their medians across the five-reservoir sample. These are sample-relative screening rules, not universal thresholds, probabilistic classifications, or transferable sedimentation criteria.

Four follow-up categories were used: (i) higher structural and dynamical signals: combined bathymetric, curve history, and operational verification; (ii) higher structural signal only: priority audit of V(H) revisions and bathymetry; (iii) higher dynamical signal only: audit of operations, reporting, gaps, and hydrometeorology; and (iv) lower signals in both layers: routine monitoring with periodic reassessment.

## 4. Results

The results follow the logic of the objectives stated in Section 1.2. Section 4.1 first verifies data support and reporting features because CECP and V(H) comparisons are meaningful only within a documented observational domain. Section 4.2 presents the temporal storage context and the structural elevation-storage layer. Section 4.3 tests whether CECP distance adds information beyond simpler descriptors. Section 4.4 and Section 4.5 report fixed-period and sliding-window CECP behavior, respectively. Section 4.6 evaluates robustness to parameter, overlap, plateau, and tie choices. Section 4.7 then converts the joint structural and dynamic evidence into engineering follow-up priorities. This organization separates computed evidence, uncertainty checks, and practical interpretation before any reservoir is assigned a monitoring recommendation.

### 4.1. Data Coverage and Reported Storage Controls

The selected records contained 2263–4586 valid paired observations and covered nominal capacities from 17.52 to 545.02 hm^3^ (Table 1). All reservoirs met the 120-observation requirement in each fixed period and supported three pairwise V(H) comparisons. The principal causal controls listed in Table 2 were not independently observed. Consequently, the results characterize reported storage behavior and operational geometry, not a closed water balance.

Exact zero increments represented 7.2–27.4% of reservoir-period observations, and 44.8–72.4% of four-point embedding vectors contained at least one tie. These frequencies confirm that reporting precision and plateaus are substantive features of the database rather than negligible numerical exceptions.

### 4.2. Temporal and Structural Storage Context

**Figure 4:**
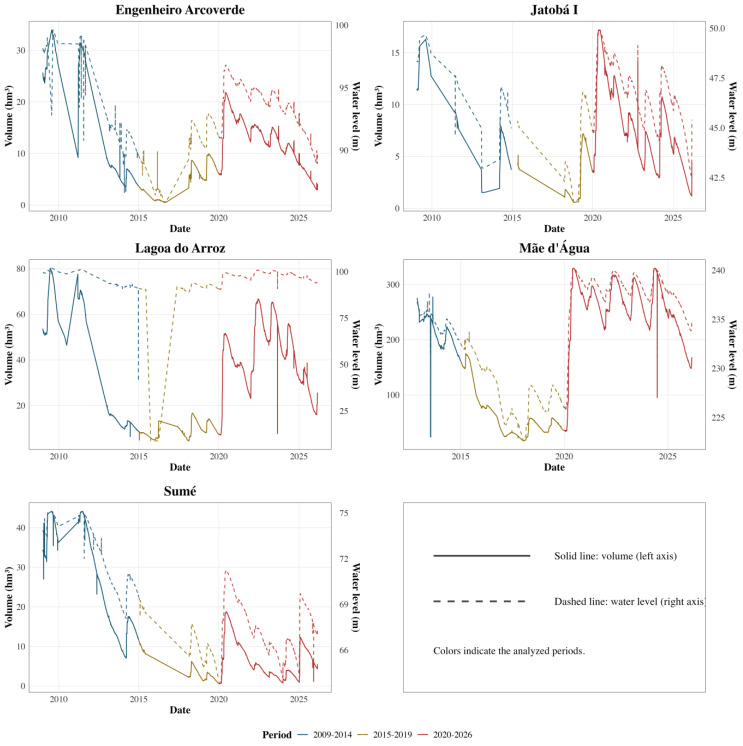
Stored volume and water-level time series. Solid lines use the left volume axis; dashed lines use the right water-level axis. Colors indicate fixed periods.

Figure 4 shows the joint volume and water-level series. Recharge and depletion amplitudes differ markedly among reservoirs, while the close co-movement of H and V confirms the expected operational dependence. The figure also shows prolonged low-storage phases and abrupt recoveries that are consistent with event-dominated semiarid hydrology, although rainfall and release records are required for attribution.

**Figure 5:**
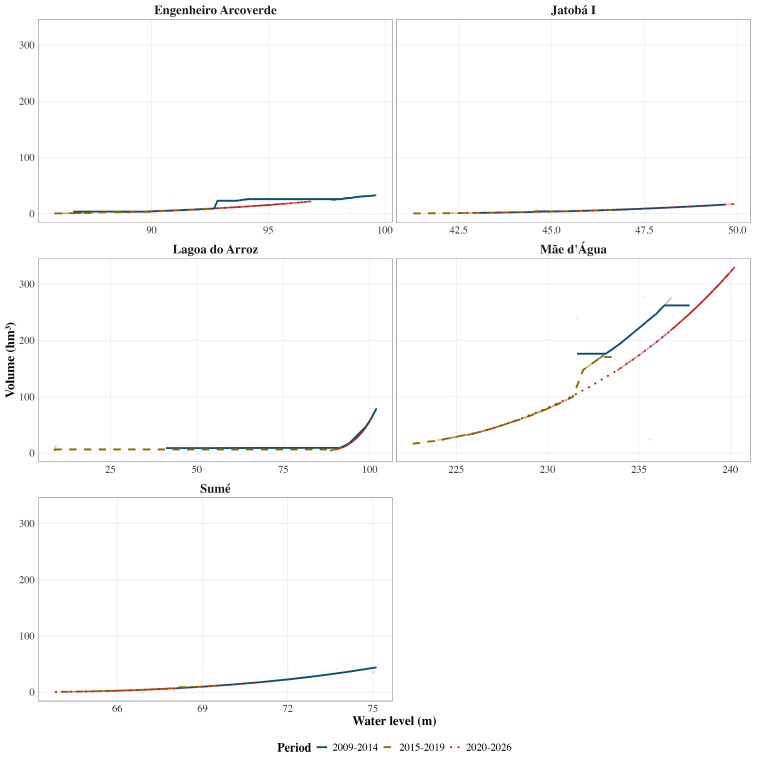
Monotone elevation-storage curves by reservoir and fixed period. Colors and line types identify the three fixed periods; the visible colored comparison boxes indicate the common water-level supports used for period-to-period curve comparison and prevent extrapolation outside shared elevation ranges.

**Figure 6:**
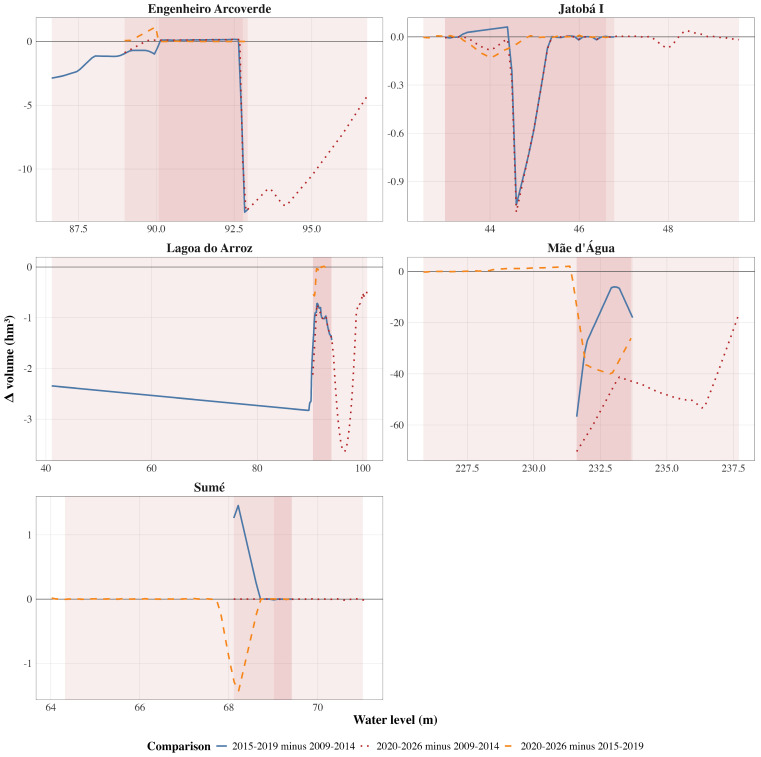
Storage differences at equivalent water levels. Red transparent regions mark elevation intervals with negative differences.

The monotone V(H) curves in Figure 5 differ across periods for all reservoirs, but the magnitude and consistency of displacement vary. Figure 6 shows the elevation-specific differences. Persistent negative segments indicate lower reported storage at equivalent elevation, whereas sign changes indicate that the relation is not a simple uniform translation.

Figure 6 isolates the storage differences at equivalent water levels and highlights the intervals where more recent periods report lower storage.

Table 3 quantifies the shifts over common elevation support. Mãe d’Água had the largest absolute long-term shift (−46.871 hm^3^; 95% CI −49.493 to −44.275 hm^3^), followed by Engenheiro Arcoverde (−5.160 hm^3^; −6.371 to −3.984 hm^3^). Relative to nominal capacity, these values represent −8.60% and −14.01%, respectively. Lagoa do Arroz showed −1.771 hm^3^ (−2.20% of capacity), Jatobá I −0.086 hm^3^ (−0.49%), and Sumé −0.001 hm^3^ (−0.003%). The narrow intervals quantify consistency over the evaluated grid; they do not account for systematic errors shared by an entire official curve.

### 4.3. Added Value Relative to Simpler Descriptors

**Figure 7:**
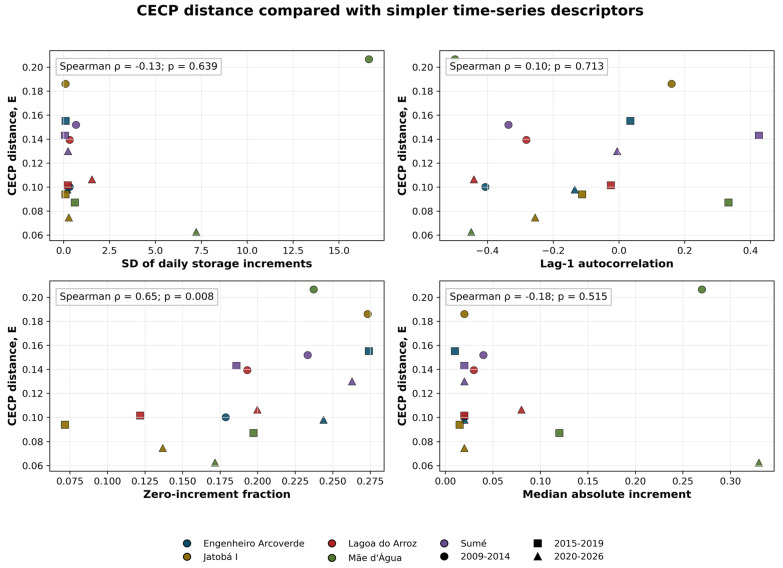
CECP distance compared with four conventional time series descriptors across 15 reservoir-period cases.

Figure 7 and Table 4 compare E with conventional statistics. The strongest associations were with the positive-increment fraction (ρ=−0.689, p=0.004) and zero-increment fraction (ρ=0.654, p=0.008). Mean increment had a moderate association (ρ=−0.514, p=0.050). In contrast, E was weakly related to standard deviation, median absolute increment, median absolute deviation, skewness, and lag-1 autocorrelation (|ρ|≤0.183).

The corresponding rank-correlation coefficients and nominal significance levels are summarized in Table 4.

The four panels clarify different aspects of this comparison. The weak association with standard deviation (ρ=−0.132, p=0.639) persists even though Mãe d’Água in 2009–2014 has a much larger increment dispersion than the remaining cases. Thus, the distance E does not simply increase with the amplitude of storage fluctuations. The near-zero association with lag-1 autocorrelation (ρ=0.104, p=0.713) likewise indicates that a pairwise linear persistence measure does not reproduce the frequency distribution of four-point ordinal patterns. Median absolute increment is also weakly associated with E (ρ=−0.183, p=0.515), showing that the typical magnitude of a reported change is distinct from its sequential ordering.

The zero-increment panel presents the clearest visible monotonic tendency among the four plotted descriptors (ρ=0.654, p=0.008). Cases with more reported plateaus tend to lie farther from (1,0) because repeated equal increments constrain the set and relative frequencies of ordinal patterns. The strongest association in Table 4 is negative for the positive-increment fraction (ρ=−0.689, p=0.004), suggesting that reservoir-periods dominated by positive reported changes tend to occupy positions closer to the maximum-entropy/minimum-complexity corner. This relationship is descriptive and may combine recharge timing, depletion sequences, operating decisions, rounding, and reporting frequency; it should not be interpreted as a causal effect of recharge or management.

These results define the incremental contribution of CECP more precisely. It is partly influenced by directional balance and reported plateaus, but it is not a proxy for increment amplitude, asymmetry, robust dispersion, or linear persistence. The ordinal distribution combines multistep order relations that no single conventional statistic in Table 4 reproduces. Conversely, the associations with zero and positive increment frequencies confirm that reporting structure and event balance remain part of the interpretation. Because the benchmark contains only 15 reservoir-period cases, the coefficients and nominal p-values are treated as descriptive evidence rather than as a definitive model-selection test, and no causal or universal threshold is inferred [25,26,27,31,32,33].

### 4.4. Fixed-Period CECP

**Figure 8:**
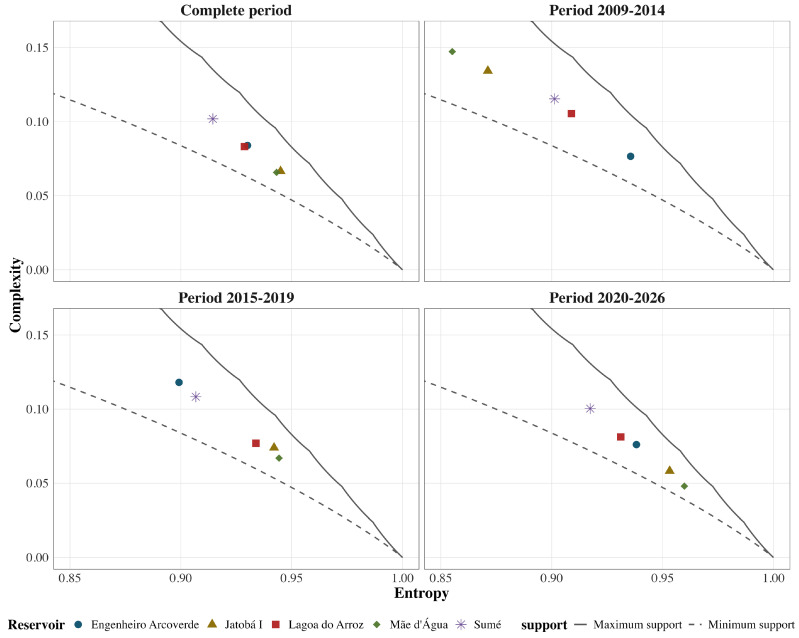
Complexity-entropy causality plane for reported daily storage increments. Gray curves show the theoretical minimum and maximum supports for D=4.

All primary CECP points occupied a high-entropy, low-to-moderate-complexity domain (Figure 8 and Table 5). In the complete record, E ranged from 0.0863 in Jatobá I to 0.1330 in Sumé. This narrow absolute range supports comparative, not categorical, interpretation.

The fixed-period coordinates and distances plotted in Figure 8 are reported in Table 5.

The temporal directions were reservoir-specific. Mãe d’Água moved from (H,C)=(0.8551,0.1473) in 2009–2014 to (0.9598,0.0480) in 2020–2026, corresponding to a marked approach toward (1,0). Jatobá I showed a similar, although smaller, movement. Engenheiro Arcoverde reached its largest E in 2015–2019, indicating an intermediate period with stronger departure from the reference corner. Lagoa do Arroz changed moderately, while Sumé retained comparatively larger E in the recent period.

### 4.5. Sliding-Window Mobility

**Figure 9:**
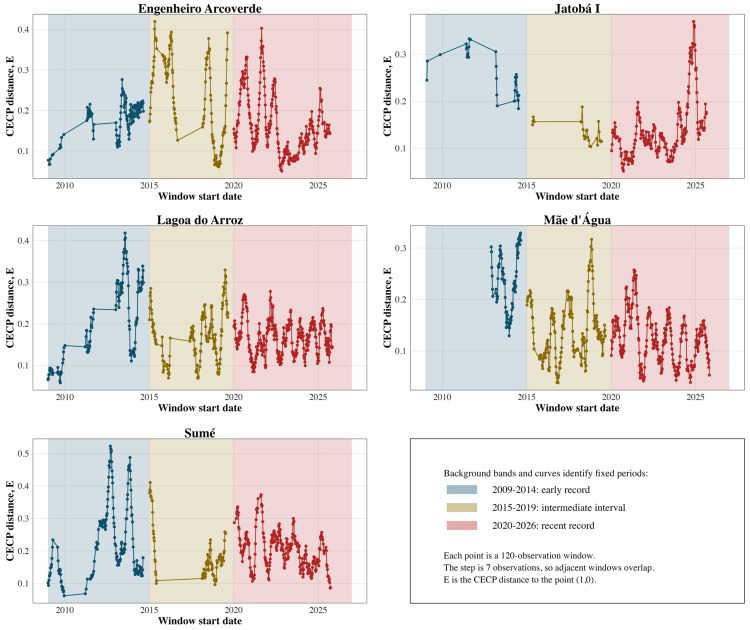
Sliding-window CECP distance using 120 observations per window and a step of seven observations. Background colors identify the fixed periods used throughout the analysis: blue for 2009–2014, ochre for 2015–2019, and red for 2020–2026. Lines and points use the same period colors. Adjacent points are dependent because windows overlap by 94.2%.

Figure 9 and Table 6 show that fixed-period coordinates conceal substantial within-period mobility. Engenheiro Arcoverde had the largest mean E in 2015–2019 (0.2354) and the largest within-period standard deviation (0.1039). Jatobá I decreased from 0.2596 to 0.1356 between the first two periods and remained near 0.1428 in 2020–2026, although its recent dispersion increased. Mãe d’Água declined progressively from 0.2321 to 0.1238. Lagoa do Arroz showed lower recent dispersion, and Sumé retained a relatively high recent mean (0.2028).

Table 6 provides the period-level mean and standard deviation of the sliding-window CECP distance for each reservoir.

The trajectories are descriptive rather than independent repeated measurements. Their value lies in locating intervals of relative movement and persistence, not in inflating the effective sample size.

### 4.6. Robustness, Reporting Artifacts, and Validation Boundary

**Figure 10:**
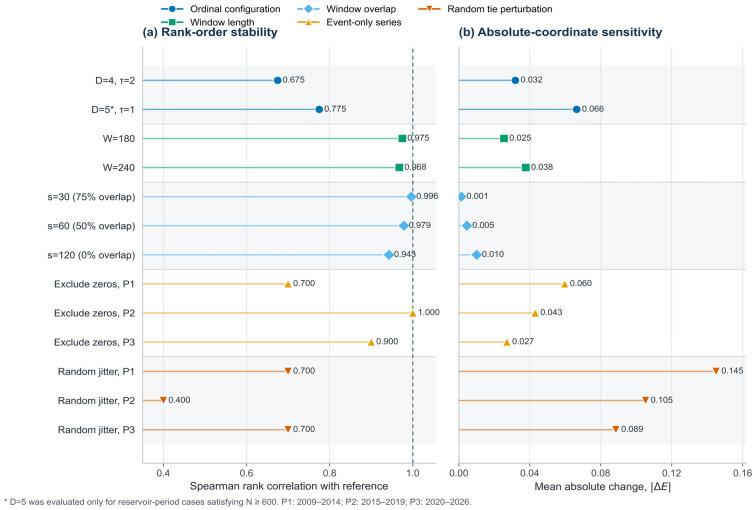
Robustness profile of CECP distance E. (**a**) Spearman rank correlation between each analytical alternative and its corresponding reference configuration. (**b**) Mean absolute change in E relative to that reference. D=5 was evaluated only for reservoir-period cases satisfying N≥600. P1: 2009–2014; P2: 2015–2019; P3: 2020–2026.

Figure 10 and Table 7 consolidate the robustness checks. The figure separates rank-order stability from absolute-coordinate sensitivity because a high rank correlation can coexist with a non-negligible displacement in E. Increasing window length preserved the reservoir-period ranking (ρ=0.975 for 180 observations and ρ=0.968 for 240) and produced only small mean absolute changes in the case-level E summaries. Reducing overlap also retained strong rank concordance: the correlation relative to step 7 was 0.996, 0.979, and 0.943 for steps 30, 60, and 120, respectively. Thus, the comparative sliding-window ordering weakened modestly but remained strongly concordant after the 94.2% overlap was removed.

Table 7 reports the numerical summaries corresponding to the robustness alternatives shown in Figure 10.

The treatment of plateaus produced the principal limitation. Excluding exact zero increments preserved period-specific rankings with ρ=0.700, 1.000, and 0.900, while mean absolute changes in E decreased from 0.060 in P1 to 0.027 in P3. In contrast, random infinitesimal jitter substantially reduced absolute E and yielded rank correlations of 0.700, 0.400, and 0.700. Random jitter converts every plateau into artificial microscopic fluctuations and is therefore an extreme boundary rather than a preferred representation. Nevertheless, it shows that absolute CECP coordinates depend on whether plateaus are retained as monitored states. For this reason, no universal E threshold is proposed, and operational recommendations are triangulated with V(H) evidence and data-quality checks.

The D=4, τ=2 sensitivity had a mean rank correlation of 0.675, whereas eligible D=5, τ=1 cases had 0.775. Parameter changes therefore preserved broad structure but not every within-period rank. This result supports the primary D=4, τ=1 configuration as the only setting uniformly compatible with the sample size rule, while preventing claims of parameter-invariant reservoir ordering.

### 4.7. Operational Prioritization

Table 8 applies the sample-relative decision matrix. Engenheiro Arcoverde and Mãe d’Água combine larger structural shifts with larger dynamical change and are the highest priorities for repeated bathymetry, official curve history verification, and review of operational records. Lagoa do Arroz has a larger structural than dynamical signal and is prioritized for V(H) audit and bathymetric verification. Jatobá I has a smaller structural shift but greater CECP change, supporting an operational, hydrometeorological, and reporting audit. Sumé has the smallest structural displacement and lower dynamical change and remains in routine monitoring.

These categories organize limited monitoring resources; they do not label reservoirs as sedimented or unsedimented. The numerical evidence should be re-evaluated when bathymetry, rating-curve documentation, or water-balance components become available.

## 5. Discussion

### 5.1. Relation to Previous Work and the Added Value of CECP

The comparison with conventional descriptors answers a central methodological question raised by the data. CECP distance is not equivalent to variance, skewness, increment magnitude, or lag-1 autocorrelation. Reservoir-period cases with similar dispersion can occupy different CECP positions because the Bandt-Pompe distribution retains the frequency of four-step order relations. This is the principal added value: a compact description of multistep ordinal organization that complements marginal, robust-scale, asymmetry, and linear persistence statistics [22,23,24,25,26,27,31,32,33].

This contribution differs from earlier hydrological entropy studies. Maximum-entropy and related approaches have been used for dependence modeling, monitoring-network design, discharge estimation, soil-moisture profiles, and river-regime characterization [12,13,14,15,16,17]. Recent uncertainty-oriented and permutation-information studies have demonstrated the usefulness of probabilistic and ordinal quantifiers for separating empirical regimes in hydrological systems and other complex signals [18,19,20,21,28,29,30]. The present analysis extends that line by combining fixed-period and moving-window CECP coordinates with an independently constructed elevation-storage layer and an explicit operational decision logic. The novelty therefore lies in the triangulated monitoring workflow, not in claiming that CECP alone measures capacity loss.

The structural component is also positioned differently from direct reservoir-sedimentation methods. Repeated bathymetry, hydrographic surveying, sediment monitoring, and calibrated remote sensing can estimate geometric or volumetric change more directly [4,5,6,7,8,9,10,11]. Operational V(H) records are less expensive and often more continuous, but they can be modified by curve revision, datum change, rounding, interpolation, or sensor practice. The observed shifts are thus best understood as screening evidence that identifies where direct methods should be concentrated. This interpretation is consistent with the broader sediment-management literature, which emphasizes that diagnosis and intervention require multiple lines of physical evidence [4,5,6,7].

The numerical scale of the present screening results is also interpretable against direct approaches, although it is not directly equivalent to them. Tessema et al. [6] and Koffi et al. [7] report physically based sedimentation assessments in which capacity loss or trapping-efficiency changes are quantified from field, bathymetric, GIS, or sedimentological information. In contrast, the present study estimates no sediment volume. Its strongest structural screening signals were relative long-term V(H) shifts of −14.01% in Engenheiro Arcoverde and −8.60% in Mãe d’Água. These values are large enough to justify engineering verification, but they remain operational curve shifts until repeated bathymetry or sediment surveys confirm the mechanism. The methodological comparison is therefore not a claim of improved accuracy over direct methods. It is a comparison of decision function: direct methods quantify capacity loss, whereas the proposed workflow ranks where those methods should be deployed first when routine monitoring data are the only continuous information source.

The contribution is not absolute. The association between E and zero or positive increment fractions shows that CECP responds to the occurrence of plateaus and directional imbalance. In an operational database, these features can be hydrologically meaningful, but they may also reflect finite precision or reporting practices. The tie and event-only analyses therefore change the interpretation from “intrinsic reservoir complexity” to “ordinal organization of the reported storage response.” This wording is more defensible and directly connected to what was measured.

The distance E is also subordinate to the full CECP coordinates. A radial distance cannot distinguish all combinations of entropy and complexity. It is useful for plotting temporal trajectories and producing a concise comparative summary, but it should not replace H and C, nor should a larger value be called more efficient, better managed, or less sedimented. The framework uses E only as a geometric monitoring coordinate.

### 5.2. Reservoir-Specific Interpretation

Engenheiro Arcoverde presents the strongest relative long-term V(H) shift (−14.01% of nominal capacity) and substantial CECP mobility. Its high 2015–2019 mean and dispersion indicate alternating reported ordinal regimes rather than a uniformly organized state. Because both layers changed, this reservoir has the strongest case for a combined investigation of bathymetry, official curve revisions, hydrological forcing, releases, and data processing.

Mãe d’Água presents the largest absolute storage shift and a long-term change equivalent to −8.60% of nominal capacity. Its fixed-period CECP trajectory moved strongly toward (1,0), while sliding-window mean E decreased through time. The convergence of structural and dynamical signals makes it a high-priority case, but the absence of repeated bathymetry prevents attribution of the V(H) shift to sedimentation alone.

Lagoa do Arroz shows a long-term structural shift of −2.20% of nominal capacity, while its CECP range and recent sliding-window dispersion are comparatively smaller. This combination is consistent with a persistent reported geometry change without equally strong dynamical mobility. The appropriate response is a targeted audit of the official V(H) history and bathymetric verification rather than a claim of dynamical degradation.

Jatobá I shows a smaller long-term V(H) shift (−0.49% of nominal capacity) but a comparatively large interperiod CECP range. The contrast implies that changes in ordinal storage dynamics can occur without a proportionally large structural displacement. Operational rules, inflow timing, reporting density, and hydrometeorological forcing should be examined before prioritizing a sediment-focused survey.

Sumé has an almost null long-term structural difference (−0.003% of nominal capacity) and the smallest interperiod E range. Its recent sliding-window dispersion is not zero, but the combined evidence places it in routine monitoring relative to the other four reservoirs. This is a comparative statement within the present sample, not proof of invariant geometry.

Taken together, these cases show why a single scalar ranking would be scientifically inadequate. Engenheiro Arcoverde and Mãe d’Água are prioritized because two conceptually distinct layers changed, whereas Jatobá I and Lagoa do Arroz illustrate discordant cases. The discordance is informative: it separates a probable need for operational or reporting investigation from a probable need for structural verification. Such separation is more useful for monitoring design than labeling every negative curve displacement as sedimentation or every CECP movement as management deterioration.

### 5.3. Joint Interpretation of Dynamic and Structural Layers

The partial decoupling between CECP and V(H) is scientifically useful. The structural layer asks whether reported volume differs at equivalent water level. The dynamic layer asks how successive storage increments are ordinally organized. A reservoir can experience a curve revision or geometric change while maintaining a relatively persistent operational response. Conversely, drought, releases, or reporting changes can alter increment patterns without shifting V(H) substantially.

For monitoring practice, a joint change in both layers supports broader and more urgent verification because multiple independent summaries changed. A structural signal without strong CECP mobility directs attention toward bathymetry and curve documentation. A dynamical signal without a structural shift directs attention toward operations, rainfall-inflow sequences, gaps, and reporting. Stability in both layers supports routine monitoring but does not eliminate the need for periodic physical surveys.

The physical meaning of the structural layer follows from $V(H)=\int_{0}^{H}A(z)\;dz$: a persistent negative ΔV(H) means that the reported integral of inundated area is smaller at the same elevation. Sediment deposition is one mechanism capable of producing that pattern, but it is not the only mechanism represented in an operational database. The physical meaning of the dynamic layer is different. CECP records how increments are ordered over short sequences, so it is sensitive to the alternation, persistence, and directional sequencing of storage changes. It does not encode the reservoir cross-section. The layers can therefore corroborate a need for investigation without being treated as measurements of the same latent quantity.

This distinction also prevents a common inferential error. A more random-like ordinal position near (1,0) is not necessarily operationally desirable, and a larger E is not necessarily evidence of instability in the engineering sense. Reservoir operation can be effective under either regular or irregular forcing. The neutral terms “ordinal organization,” “temporal mobility,” and “departure from the reference corner” are therefore used instead of value-laden labels such as efficiency, predictability, or degradation.

### 5.4. Robustness and Generalization

Window overlap was not responsible for the comparative ranking because nonoverlapping windows retained a high rank correlation with the primary trajectory summaries. Larger windows also preserved ranks while reducing dispersion, as expected from smoothing. These results support the use of the seven-observation step for temporal resolution, provided that adjacent points are explicitly treated as dependent.

The overlap experiment is important because a dense moving-window trajectory can create the visual impression of abundant independent evidence. Here, reducing overlap from 94.2% to zero retained strong concordance in the case ranking (ρ=0.943). This does not make adjacent primary windows independent; rather, it shows that the reservoir-period ordering was not generated solely by repeated reuse of the same observations. The principal trajectory is retained for temporal localization, while nonoverlapping windows provide the more conservative robustness reference.

Embedding and delay sensitivities were less uniform. The primary setting is justified by complete sample eligibility and established ordinal practice, not by an assertion that it is uniquely correct. The frequent ties are a more consequential limitation. Event-only analysis preserved much of the period ranking, whereas random jitter demonstrated that the absolute distance from (1,0) is strongly affected when plateaus are transformed into noise. Future studies should compare deterministic, tie-aware, weighted, and amplitude-sensitive ordinal methods and should report measurement resolution alongside CECP coordinates [25,26,27].

The difference between the event-only and jitter experiments clarifies the role of plateaus. Removing zero increments asks how ordinal organization changes when attention is restricted to reported storage events. Random jitter asks what would happen if every plateau were resolved into arbitrary microscopic order. The first has an operational interpretation; the second is a deliberately adverse numerical perturbation. Their different effects show that ties cannot be dismissed as a minor computational detail, but neither should random tie breaking be adopted as a physically neutral correction.

The five reservoirs belong to a restricted semiarid setting and were selected for data eligibility. The findings cannot be generalized to humid climates, snowmelt systems, different reservoir morphometries, or contrasting operating rules. Transferability requires replication with documented water balances, repeated bathymetry, and independent curve histories.

### 5.5. External Validation and Causal Limits

The study does not contain external bathymetric validation. Therefore, it cannot estimate sedimentation rate, dead-storage growth, diagnostic sensitivity, specificity, or predictive error. Bootstrap intervals quantify variability across the common elevation grid but do not include shared systematic errors. Similarly, CECP robustness tests quantify computational dependence on analytical choices but do not identify physical drivers.

The next validation stage should pair operational records with at least two bathymetric surveys, metadata on official elevation-storage revisions, precipitation and evaporation, inflow and release series, and sediment-yield or concentration measurements. A prospective test could define the present workflow before observing the later bathymetric change and then assess whether screening priority predicts independently measured capacity loss. Until such evidence exists, the framework should be described as exploratory monitoring and prioritization.

### 5.6. Implications for Monitoring Design

The practical advantage of the framework is not the replacement of field campaigns but the allocation of scarce monitoring effort. Comprehensive bathymetric surveys are technically preferable for quantifying capacity change, yet they are costly and are rarely repeated at the temporal frequency of operational records [8,9,10,11]. A reproducible screening layer can identify reservoirs for which curve history retrieval, instrument checks, water-balance reconstruction, or bathymetry would have the highest immediate information value.

For the present sample, the recommended sequence is hierarchical. First, agencies should verify the provenance and revision history of the operational V(H) curves. Second, reservoirs with convergent structural and dynamic signals should receive bathymetric and operational investigation. Third, discordant cases should be examined according to the layer that changed. Finally, CECP and V(H) summaries should be recalculated after metadata or physical measurements become available. This iterative use is compatible with adaptive reservoir governance in the Brazilian semiarid region [38], while remaining explicitly subordinate to direct physical evidence.

## 6. Conclusions

This study presents a transparent information-theoretic workflow for screening reported storage dynamics in data-limited semiarid reservoirs. CECP quantifiers captured multistep ordinal organization that was weakly related to variance, skewness, increment magnitude, and lag-1 autocorrelation, although they were associated with zero and positive increment frequencies. This complementary role is consistent with the distinction between ordinal-pattern quantifiers and conventional distributional or linear descriptors [22,23,24,25,26,27,31,32,33]. Sliding-window rankings remained stable when overlap was reduced or removed, supporting their comparative use as dependent descriptive trajectories.

Monotone V(H) comparisons identified the largest relative long-term shifts in Engenheiro Arcoverde, Mãe d’Água, and Lagoa do Arroz. These operational shifts are compatible with storage geometry change but are not unique evidence of sedimentation. Their interpretation as screening evidence, followed by bathymetric or hydrographic verification, is supported by established reservoir capacity assessment practice [4,5,6,7,8,9,10,11]. Combining structural magnitude, CECP change, and data-quality diagnostics prioritized Engenheiro Arcoverde and Mãe d’Água for comprehensive verification, Lagoa do Arroz for structural audit, Jatobá I for dynamical and operational audit, and Sumé for routine reassessment.

The central limitation is the absence of repeated bathymetry and water-balance components. Ties and reported plateaus also affect absolute CECP coordinates, as anticipated by the permutation entropy literature on finite samples, ties, and amplitude information [25,26,27,35]. Accordingly, the method does not diagnose sedimentation and proposes no universal threshold. Its contribution is a reproducible way to organize incomplete evidence, identify where different monitoring layers agree or diverge, and allocate follow-up investigation more rationally. Independent validation should combine repeated bathymetry, documented rating-curve histories, and reservoir-specific water-balance and sediment observations before the framework is used beyond exploratory prioritization [4,5,6,7,8,9,10,11].

Future work should test the workflow in reservoirs with repeated bathymetric campaigns, documented curve revisions, rainfall, inflow, evaporation, release, and sediment concentration records. A prospective design should define CECP and V(H) screening rules before the next bathymetric survey and then compare the ranking with independently measured storage loss. Replication in humid, snowmelt-dominated, regulated hydropower, and multi-reservoir cascade systems is also required before any generalized threshold can be proposed.

## Figures and Tables

**Table 1:** Characteristics and empirical coverage of the five selected reservoirs.

Reservoir	Municipality	Hydrographic System	Capacity (hm^3^)	Monitoring Record	Paired H-V	H Range (m)	V Range (hm^3^)
Engenheiro Arcoverde	Condado	Piancó-Piranhas-Açu	36.83	6 January 2009 to 2 March 2026	3884	85.84–99.63	0.52–33.98
Jatobá I	Patos	Piancó-Piranhas-Açu (Espinharas sub-basin)	17.52	30 January 2009 to 2 March 2026	2263	41.26–49.92	0.57–17.21
Lagoa do Arroz	Cajazeiras	Piancó-Piranhas-Açu (Rio do Peixe sub-basin)	80.39	1 January 2009 to 2 March 2026	4586	8.86–101.89	4.49–80.22
Mãe d’Água	Coremas	Piancó-Piranhas-Açu	545.02	22 November 2012 to 2 March 2026	4269	222.61–240.22	16.54–329.72
Sumé	Sumé	Paraíba River (Upper Paraíba sub-basin)	44.86	2 January 2009 to 2 March 2026	4113	63.79–75.12	0.55–44.16

**Table 2:** Principal controls of reservoir storage and their availability in the present database.

Control	Expected Storage Effect	Evidence Used Here	Reservoir-Specific Series Available
Rainfall and drought	Event-driven recharge and prolonged depletion	Regional semiarid and drought literature	No
Inflow and catchment runoff	Rapid positive storage increments after runoff events	Represented only indirectly in observed storage changes	No
Evaporation	Persistent dry-season storage loss	Regional process knowledge	No
Releases and withdrawals	Negative increments and operational regime shifts	Represented only indirectly in observed storage changes	No
Storage geometry and sediment accumulation	Change in V(H) at equivalent water levels	Operational H-V pairs and monotone V(H) reconstructions	H-V only; no repeated bathymetry
Monitoring and reporting	Ties, rounding, gaps, and artificial zero increments	Dates, reported H and V precision, and tie diagnostics	Yes

**Table 3:** Mean storage differences and bootstrap uncertainty over the common water-level grid.

Reservoir	Comparison	H Range (m)	n Levels	Mean ΔV (hm^3^)	95% CI (hm^3^)	*P* (ΔV < 0)	Bootstrap *p*
Engenheiro Arcoverde	2015–2019 vs. 2009–2014	86.64 to 92.94	64	−1.265	−1.928 to −0.748	1.0	<0.001
Engenheiro Arcoverde	2020–2026 vs. 2015–2019	88.98 to 92.88	40	0.142	0.055 to 0.244	0.0	<0.001
Engenheiro Arcoverde	2020–2026 vs. 2009–2014	88.98 to 96.78	79	−5.16	−6.371 to −3.984	1.0	<0.001
Jatobá I	2015–2019 vs. 2009–2014	42.99 to 46.79	39	−0.118	−0.214 to −0.036	0.999	0.002
Jatobá I	2020–2026 vs. 2015–2019	42.51 to 46.70	43	−0.024	−0.037 to −0.013	1.0	<0.001
Jatobá I	2020–2026 vs. 2009–2014	42.99 to 49.59	67	−0.086	−0.146 to −0.036	1.0	<0.001
Lagoa do Arroz	2015–2019 vs. 2009–2014	41.09 to 94.09	531	−2.478	−2.513 to −2.441	1.0	<0.001
Lagoa do Arroz	2020–2026 vs. 2015–2019	90.53 to 94.03	36	−0.101	−0.168 to −0.044	1.0	<0.001
Lagoa do Arroz	2020–2026 vs. 2009–2014	90.53 to 100.83	104	−1.771	−1.982 to −1.569	1.0	<0.001
Mãe d’Água	2015–2019 vs. 2009–2014	231.61 to 233.71	22	−19.519	−25.390 to −14.288	1.0	<0.001
Mãe d’Água	2020–2026 vs. 2015–2019	225.85 to 233.65	79	−8.679	−12.351 to −5.376	1.0	<0.001
Mãe d’Água	2020–2026 vs. 2009–2014	231.61 to 237.71	62	−46.871	−49.493 to −44.275	1.0	<0.001
Sumé	2015–2019 vs. 2009–2014	68.11 to 69.41	14	0.395	0.138 to 0.706	0.001	0.002
Sumé	2020–2026 vs. 2015–2019	64.02 to 69.42	55	−0.131	−0.226 to −0.051	1.0	<0.001
Sumé	2020–2026 vs. 2009–2014	68.11 to 71.11	31	−0.001	−0.003 to −0.000	0.977	0.046

**Table 4:** Spearman association between CECP distance E and conventional descriptors.

Simple Descriptor	Spearman ρ with E	Two-Sided *p*-Value
Positive-increment fraction	−0.689	0.004
Zero-increment fraction	0.654	0.008
Mean δV (hm^3^ day^−1^)	−0.514	0.050
Median |δV| (hm^3^ day^−1^)	−0.183	0.515
SD δV (hm^3^ day^−1^)	−0.132	0.639
Lag-1 autocorrelation	0.104	0.713
Skewness	−0.089	0.752
MAD δV (hm^3^ day^−1^)	0.048	0.864

**Table 5:** Entropy, statistical complexity, and geometric CECP distance for fixed periods.

Ranking	Reservoir	Period	Entropy	Complexity	Dist. to (1, 0)
1	Sumé	Complete period	0.9144	0.1019	0.1330
2	Lagoa do Arroz	Complete period	0.9287	0.0832	0.1095
3	Engenheiro Arcoverde	Complete period	0.9301	0.0840	0.1093
4	Mãe d’Água	Complete period	0.9432	0.0658	0.0869
5	Jatobá I	Complete period	0.9450	0.0666	0.0863
1	Mãe d’Água	2009–2014	0.8551	0.1473	0.2066
2	Jatobá I	2009–2014	0.8712	0.1343	0.1861
3	Sumé	2009–2014	0.9013	0.1154	0.1519
4	Lagoa do Arroz	2009–2014	0.9089	0.1054	0.1394
5	Engenheiro Arcoverde	2009–2014	0.9356	0.0766	0.1001
1	Engenheiro Arcoverde	2015–2019	0.8992	0.1180	0.1552
2	Sumé	2015–2019	0.9068	0.1083	0.1429
3	Lagoa do Arroz	2015–2019	0.9339	0.0770	0.1015
4	Jatobá I	2015–2019	0.9420	0.0739	0.0939
5	Mãe d’Água	2015–2019	0.9443	0.0669	0.0871
1	Sumé	2020–2026	0.9173	0.1003	0.1300
2	Lagoa do Arroz	2020–2026	0.9311	0.0812	0.1065
3	Engenheiro Arcoverde	2020–2026	0.9382	0.0761	0.0980
4	Jatobá I	2020–2026	0.9532	0.0582	0.0747
5	Mãe d’Água	2020–2026	0.9598	0.0480	0.0626

**Table 6:** Sliding-window CECP distance by reservoir and fixed period.

Reservoir	Period	N Windows	Mean CECP Distance E	SD CECP Distance E
Engenheiro Arcoverde	2009–2014	114	0.1804	0.0432
Engenheiro Arcoverde	2015–2019	130	0.2354	0.1039
Engenheiro Arcoverde	2020–2026	249	0.1700	0.0737
Jatobá I	2009–2014	33	0.2596	0.0468
Jatobá I	2015–2019	25	0.1356	0.0224
Jatobá I	2020–2026	208	0.1428	0.0680
Lagoa do Arroz	2009–2014	136	0.2082	0.0969
Lagoa do Arroz	2015–2019	157	0.1706	0.0606
Lagoa do Arroz	2020–2026	299	0.1670	0.0410
Mãe d’Água	2009–2014	82	0.2321	0.0548
Mãe d’Água	2015–2019	188	0.1395	0.0574
Mãe d’Água	2020–2026	278	0.1238	0.0482
Sumé	2009–2014	170	0.2334	0.1158
Sumé	2015–2019	88	0.1842	0.0748
Sumé	2020–2026	267	0.2028	0.0583

**Table 7:** Summary of CECP robustness checks.

Robustness Check	Alternative	Coverage	Main Result
Embedding/delay	D = 4, τ = 2	20 cases	Mean |ΔE| = 0.032; mean rank ρ = 0.675
Embedding/delay	D = 5, τ = 1	18 cases	Mean |ΔE| = 0.066; mean rank ρ = 0.775
Window length	180 observations	2291 windows	Rank ρ vs. 120 = 0.975
Window length	240 observations	2162 windows	Rank ρ vs. 120 = 0.968
Window overlap	Step 30; 75.0% overlap	571 windows	Rank ρ vs. step 7 = 0.996
Window overlap	Step 60; 50.0% overlap	290 windows	Rank ρ vs. step 7 = 0.979
Window overlap	Step 120; 0.0% overlap	148 windows	Rank ρ vs. step 7 = 0.943
Reported plateaus	Exclude exact zeros, 2009–2014	mean zero fraction = 0.223	rank ρ = 0.700; mean |ΔE| = 0.060
Reported plateaus	Exclude exact zeros, 2015–2019	mean zero fraction = 0.170	rank ρ = 1.000; mean |ΔE| = 0.043
Reported plateaus	Exclude exact zeros, 2020–2026	mean zero fraction = 0.203	rank ρ = 0.900; mean |ΔE| = 0.027
Extreme tie perturbation	Random infinitesimal jitter, 2009–2014	mean tied-vector fraction = 0.665	rank ρ = 0.700; mean |ΔE| = 0.145
Extreme tie perturbation	Random infinitesimal jitter, 2015–2019	mean tied-vector fraction = 0.610	rank ρ = 0.400; mean |ΔE| = 0.105
Extreme tie perturbation	Random infinitesimal jitter, 2020–2026	mean tied-vector fraction = 0.614	rank ρ = 0.700; mean |ΔE| = 0.089

**Table 8:** Sample-relative screening matrix and recommended follow-up.

Reservoir	Long-Term ΔV (% Capacity)	Interperiod E Range	Recent SD E	Recommended Follow-Up
Engenheiro Arcoverde	−14.01	0.057	0.074	Highest priority: verify bathymetry, curve history, and operations
Jatobá I	−0.49	0.111	0.068	Dynamical priority: audit operations, gaps, and hydrometeorology
Lagoa do Arroz	−2.20	0.038	0.041	Structural priority: audit V(H) revisions and plan bathymetry
Mãe d’Água	−8.60	0.144	0.048	Highest priority: verify bathymetry, curve history, and operations
Sumé	−0.00	0.022	0.058	Routine monitoring with periodic reassessment

## Data Availability

The operational data were obtained from AESA. Processed data, complete analytical outputs, and scripts are available from the corresponding author upon reasonable request.

## Nomenclature

AESA	Executive Water Management Agency of the State of Paraíba
CECP	Complexity–entropy causality plane
H	Water level in the elevation-storage context; normalized permutation entropy when written as H[P]
V	Stored volume
V(H)	Elevation-storage relation
$\Delta V_{t}$	Storage change between two consecutive reports
$\delta V_{t}$	Daily normalized storage increment
D	Embedding dimension
τ	Ordinal delay
$P=\{p_{i}\}$	Bandt-Pompe ordinal-pattern probability distribution
H[P]	Normalized permutation entropy
C[P]	Martín-Plastino-Rosso statistical complexity
E	Euclidean distance from the CECP point (H,C) to (1,0)
W	Sliding-window length in observations
s	Sliding-window step in observations
O(W,s)	Percentage overlap between consecutive sliding windows
$f_{0}$	Fraction of exact zero or numerically zero storage increments
$f_{+}$	Fraction of positive storage increments
$f_{tie}$	Fraction of embedding vectors containing tied values
$S_{r}$	Sample-relative structural magnitude for reservoir r
$R_{r}$	Interperiod CECP distance range for reservoir r
$M_{r}$	Recent sliding-window mobility for reservoir r

## Appendix A

Appendix A reports supporting checks used to document data eligibility, compare conventional descriptors with the CECP analysis, and quantify sensitivity to tied ordinal patterns. These tables are provided as supplementary evidence for reproducibility and for separating data support issues from the main interpretation.

Table A1 documents the reservoir selection criteria, including valid paired observations, common elevation support, and whether each reservoir satisfied the minimum sample requirement for all three fixed periods.

**Table A1:** Reservoir eligibility and common-support audit.

Reservoir	Record	P1	P2	P3	Common Support V(H)	Decision
Acauã	2013 to 2026	0	700	2219	1/3	Excluded
Araçagi	2013 to 2026	0	651	1756	1/3	Excluded
Engenheiro Arcoverde	2009 to 2026	953	1068	1863	3/3	Included
Epitácio Pessoa	2009 to 2026	51	1048	2215	1/3	Excluded
Farinha	2013 to 2026	0	61	1535	0/3	Excluded
Jatobá I	2009 to 2026	349	317	1597	3/3	Included
Lagoa do Arroz	2009 to 2026	1114	1259	2213	3/3	Included
Mãe d’Água	2012 to 2026	690	1479	2100	3/3	Included
Piranhas	2013 to 2026	0	232	1554	1/3	Excluded
Sumé	2009 to 2026	1348	738	2027	3/3	Included

Table A2 summarizes conventional distributional and linear descriptors of the daily storage increments. These values provide the baseline against which the information-theoretic CECP distance was compared in the main text.

**Table A2:** Conventional descriptors of daily storage increments.

Reservoir	Period	N	Mean δV (hm^3^ day^−1^)	SD δV (hm^3^ day^−1^)	Lag-1 Autocorrelation	Zero-Increment Fraction	Positive-Increment Fraction
Engenheiro Arcoverde	2009–2014	912	−0.005	0.330	−0.406	0.179	0.083
Engenheiro Arcoverde	2015–2019	1029	−0.002	0.111	0.035	0.274	0.093
Engenheiro Arcoverde	2020–2026	1862	−0.000	0.199	−0.135	0.244	0.126
Jatobá I	2009–2014	344	−0.000	0.100	0.160	0.273	0.087
Jatobá I	2015–2019	293	0.000	0.106	−0.112	0.072	0.150
Jatobá I	2020–2026	1579	0.004	0.282	−0.255	0.137	0.121
Lagoa do Arroz	2009–2014	1067	−0.028	0.332	−0.282	0.193	0.100
Lagoa do Arroz	2015–2019	1215	0.004	0.228	−0.024	0.122	0.114
Lagoa do Arroz	2020–2026	2212	0.009	1.547	−0.442	0.200	0.158
Mãe d’Água	2009–2014	687	−0.172	16.630	−0.499	0.237	0.099
Mãe d’Água	2015–2019	1435	−0.036	0.615	0.333	0.197	0.126
Mãe d’Água	2020–2026	2063	0.075	7.222	−0.449	0.172	0.186
Sumé	2009–2014	1303	−0.017	0.673	−0.336	0.233	0.071
Sumé	2015–2019	737	−0.006	0.071	0.425	0.186	0.077
Sumé	2020–2026	1986	0.004	0.241	−0.006	0.263	0.096

Table A3 reports the sensitivity of fixed-period CECP estimates to random resolution of tied ordinal patterns. It is used to identify cases in which reported plateaus can affect absolute CECP coordinates.

**Table A3:** Sensitivity of fixed-period CECP estimates to random tie resolution.

Reservoir	Period	Tied-Vector Fraction	Baseline E	Random Tie Mean E	Random Tie SD E
Engenheiro Arcoverde	2009–2014	0.648	0.100	0.007	0.002
Engenheiro Arcoverde	2015–2019	0.707	0.155	0.014	0.003
Engenheiro Arcoverde	2020–2026	0.633	0.098	0.007	0.002
Jatobá I	2009–2014	0.622	0.186	0.018	0.006
Jatobá I	2015–2019	0.448	0.094	0.018	0.005
Jatobá I	2020–2026	0.459	0.075	0.006	0.001
Lagoa do Arroz	2009–2014	0.668	0.139	0.008	0.002
Lagoa do Arroz	2015–2019	0.595	0.101	0.008	0.002
Lagoa do Arroz	2020–2026	0.681	0.106	0.005	0.001
Mãe d’Água	2009–2014	0.675	0.207	0.010	0.004
Mãe d’Água	2015–2019	0.590	0.087	0.005	0.002
Mãe d’Água	2020–2026	0.575	0.063	0.003	0.001
Sumé	2009–2014	0.713	0.152	0.016	0.003
Sumé	2015–2019	0.711	0.143	0.010	0.003
Sumé	2020–2026	0.724	0.130	0.009	0.002
